# 2073: Fecal bile acids, fecal short-chain fatty acids and the intestinal microbiota in patients with irritable bowel syndrome (IBS) and control volunteers

**DOI:** 10.1017/cts.2017.115

**Published:** 2018-05-10

**Authors:** Andrea Shin, David Nelson, John Wo, Michael Camilleri, Anita Gupta, Arturo Carrillo, Huiping Xu

## Abstract

OBJECTIVES/SPECIFIC AIMS: Recent data suggest that fecal microbiota and intraluminal organic acids may play an important role in irritable bowel syndrome (IBS) pathogenesis through effects on intestinal secretion and motility. Understanding their contribution will be critical in developing diagnostic and treatment strategies. Objectives and goals of this study will be to: (1) compare fecal microbiota and fecal organic acids in IBS patients and controls and (2) investigate the association between colonic transit and fecal microbiota in IBS patients and controls. METHODS/STUDY POPULATION: We propose a prospective investigation of fecal organic acids, colonic transit and fecal microbiota in 36 IBS patients and 18 healthy controls. The target population will be adults ages 18–65 years meeting Rome IV criteria for IBS (both diarrhea predominant and constipation-predominant, IBS-D, and IBS-C) and asymptomatic controls. Exclusion criteria are: (a) history of microscopic colitis, inflammatory bowel disease, celiac disease, cancer, chronic infectious disease, immunodeficiency, uncontrolled thyroid disease, liver disease, or elevated AST/ALT>2.0x the upper limit of normal, (b) prior radiation therapy of the abdomen or abdominal surgeries with the exception of appendectomy or cholecystectomy>6 months before study initiation, (c) ingestion of prescription, over the counter, or herbal medications affecting gastrointestinal transit or study interpretation within 6 months of study initiation for controls or within 2 days before study initiation for IBS patients, (d) pregnant females, (e) antibiotic usage within 3 months prior to study participation, (f) prebiotic or probiotic usage within the 2 weeks prior to study initiation, (g) tobacco users. Primary outcomes will be fecal bile acid excretion and profile, short-chain fatty acid (SCFA) excretion and profile, colonic transit, and fecal microbiota. Secondary outcomes will be stool characteristics based on responses to validated bowel diaries. Stool samples will be collected from participants during the last 2 days of a 4-day 100-g fat diet and split into 3 samples for fecal microbiota, SCFA, and bile acid analysis and frozen. Frozen aliquots will be shipped to the Metabolite Profiling Facility at Purdue University and the Mayo Clinic Department of Laboratory Medicine and Pathology for SCFA and bile acid measurements, respectively. Analysis of fecal microbiota will be performed in the research laboratory of Dr. David Nelson in collaboration with bioinformatics expertise affiliated with the Nelson lab. Colonic transit time will be measured with the previously validated method using radio-opaque markers. Generalized linear models will be used as the analysis framework for comparing study endpoints among groups. RESULTS/ANTICIPATED RESULTS: This study seeks to examine the innovative concept that specific microbial signatures are associated with increased fecal excretion of organic acids to provide unique insights on a potential mechanistic link between altered intraluminal organic acids and fecal microbiota. DISCUSSION/SIGNIFICANCE OF IMPACT: Results may lead to development of targets for novel therapies and diagnostic biomarkers for IBS, emphasizing the role of the fecal metabolome.

